# Aggressiveness of human melanoma xenograft models is promoted by aneuploidy-driven gene expression deregulation

**DOI:** 10.18632/oncotarget.473

**Published:** 2012-04-24

**Authors:** Véronique Mathieu, Christine Pirker, Wolfgang M. Schmidt, Sabine Spiegl-Kreinecker, Daniela Lötsch, Petra Heffeter, Balazs Hegedus, Michael Grusch, Robert Kiss, Walter Berger

**Affiliations:** ^1^ Laboratory of Toxicology, Faculty of Pharmacy, Université Libre de Bruxelles, Brussels, Belgium; ^2^ Department of Medicine I, Institute of Cancer Research, Medical University Vienna, Vienna, Austria; ^3^ Comprehensive Cancer Center, Medical University Vienna, Vienna, Austria; ^4^ Neuromuscular Research Department, Center for Anatomy and Cell Biology, Medical University Vienna, Vienna, Austria; ^5^ Department of Neurosurgery, Landesnervenklinik Wagner-Jauregg Hospital, Linz, Austria; ^6^ Department of Thoracic Surgery, Medical University Vienna, Vienna, Austria; ^7^ 2nd Institute of Pathology, Semmelweis University, Budapest, Hungary

**Keywords:** malignant melanoma, aneuploidy, local aggressiveness, xenograft, integrative genomics

## Abstract

Melanoma is a devastating skin cancer characterized by distinct biological subtypes. Besides frequent mutations in growth- and survival-promoting genes like *BRAF* and *NRAS*, melanomas additionally harbor complex non-random genomic alterations. Using an integrative approach, we have analysed genomic and gene expression changes in human melanoma cell lines (N=32) derived from primary tumors and various metastatic sites and investigated the relation to local growth aggressiveness as xenografts in immuno-compromised mice (N=22). Although the vast majority (>90%) of melanoma models harbored mutations in either *BRAF* or *NRAS*, significant differences in subcutaneous growth aggressiveness became obvious. Unsupervised clustering revealed that genomic alterations rather than gene expression data reflected this aggressive phenotype, while no association with histology, stage or metastatic site of the original melanoma was found. Genomic clustering allowed separation of melanoma models into two subgroups with differing local growth aggressiveness *in vivo*. Regarding genes expressed at significantly altered levels between these subgroups, a surprising correlation with the respective gene doses (>85% accordance) was found. Genes deregulated at the DNA and mRNA level included well-known cancer genes partly already linked to melanoma (*RAS* genes, *PTEN*, *AURKA*, *MAPK* inhibitors Sprouty/Spred), but also novel candidates like *SIPA1* (a Rap1GAP). Pathway mining further supported deregulation of Rap1 signaling in the aggressive subgroup e.g. by additional repression of two Rap1GEFs. Accordingly, siRNA-mediated down-regulation of SIPA1 exerted significant effects on clonogenicity, adherence and migration in aggressive melanoma models. Together our data suggest that an aneuploidy-driven gene expression deregulation drives local aggressiveness in human melanoma.

## INTRODUCTION

Melanomas account for 4 to 5% of all cancers and represent currently the 6^th^ leading cancer type in the USA [1]. While 80% of melanomas are diagnosed at localized stages, one third of those early stage patients will develop metastatic disease associated with dismal prognosis, i.e. a median overall survival of 6 to 8 months [2]. Recent discoveries have markedly improved our understanding of the molecular changes underlying malignant progression of melanomas including mainly alterations in proliferation, survival and cell death signaling pathways [3-5]. The RAF/MEK/ERK and PI3K/AKT are two major signaling pathways constitutively activated in up to 90% of melanomas. While *BRAF* mutations represent the most frequent oncogenic alteration in melanomas so far (*BRAF^V600E^* in up to 70% of cases), *NRAS* mutations occur in 15 to 30%. Recently, exon sequencing approaches revealed additional mutations in individual members of the MAP3K and MAP2K families including *MEK1* and *MEK2*. The AKT/mTOR pathway might be additionally activated mainly by loss-of-function mutations or deletions of the inhibiting phosphatase *PTEN*. Furthermore, typical impairment of senescence due to mutations, deletions or methylation of *p16^INK4/CDKN2A^* occurs in 30 to 70% of melanomas. Oncogenic proteins in melanoma include e.g. members of the bcl-2 protein family, cyclin D1, and several transcription factors like the lineage-specific oncogene MITF (for detailed reviews on these molecular changes see [6-8]).

Improving the knowledge on major drivers underlying development and aggressiveness of melanoma is of strong interest to identify clinically and therapeutically relevant patient subgroups. However, achievement of this goal is hampered by strong heterogeneity not only at the genomic level, but also with regard to phenotypic, histopathological, and clinical characteristics. Accordingly, multiple studies from different scientific disciplines have suggested the existence of several melanoma subtypes that may arise through several different causative pathways [9]. At the molecular level, besides (in)activating mutations in proto-oncogenes and tumor suppressor genes, development of melanoma is characterized by complex karyotypic changes leading to multiple and severe gene dose alterations. Several lines of evidence suggest that this aneuploidy might represent an additional driving force of malignant transformation and cancer progression [7, 9]. It can be assumed that the observed molecular heterogeneity drives at least to some extent disease pathogenesis, clinical behavior, and possibly response to therapy, and that genomic aberrations and gene dose-related RNA alteration patterns might even dictate disease behavior [7]. Accordingly, clustering of 80 metastatic lesions based on genomic alteration profiles resulted in three subgroups that could not be related to their location, but, when intersected with clinical outcome, one subgroup displayed a significant survival advantage, indicating that the clustering could be biologically relevant [7].

In this study, we aimed to analyze genomic and transcriptomic alterations in human melanoma cell cultures (originating from primary as well as metastatic lesions) classified with respect to their *in vivo* growth characteristics. Using this approach, we demonstrated that genomic aberrations allow clustering of primary melanoma cell lines according to their *in vivo* growth behavior. Interestingly, genes differentially expressed in subgroups with differing aggressiveness closely reflected corresponding gene dose alterations. This suggests that melanoma malignancy is at least partly driven by aneuploidy-mediated gene expression deregulation. The affected genes comprised several known oncogenes and tumor-suppressors. However, also novel candidates like *SIPA1*, a Rap1GTPase, were identified as important drivers of melanoma aggressiveness. Accordingly, by using a siRNA approach, this Rap1-deactivating protein was proven to regulate clonogenicity and cell adhesion/migration of aggressive melanoma cell models.

## RESULTS

### Human melanoma xenograft models are characterized by distinctly differing aggressiveness

**Figure 1:**
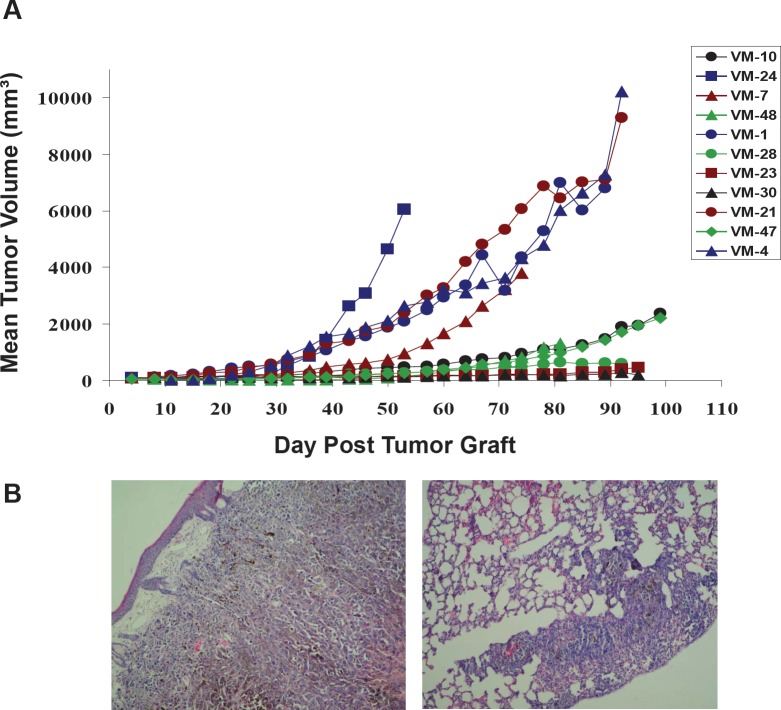
Subcutaneous growth of the indicated 11 human melanoma cell models in immuno-compromised mice (A) As described in Material and Methods, 2.5×10^6^ melanoma cells were xenografted into immuno-compromised mice and local tumor growth was measured at the indicated time points. (B) Representative H&E staining of the fast-growing melanoma xenograft model VM-21 characterized by invasive growth subcutaneously (left) and causing lung metastasis (right panel).

In an initial approach, 11 human cutaneous melanoma cell models were xenografted subcutaneously into immuno-compromised nu/nu mice and primary tumor growth as well as metastasis to the lungs, the liver and the brain investigated. While all cell lines were tumorigenic, distinct differences in aggressiveness became obvious (Figure 1A). Five cell models were characterized by rapid growth with two showing spontaneous metastasis to the lung (Figure 1B). In contrast, the other 6 melanoma models, though all tumorigenic, were much less aggressive and no signs of local or distant metastases could be detected. Growth behaviour was confirmed in the SCID mouse system (data not shown). Tumorigenicity *in vivo* did not reflect the growth characteristics of the cell models *in vitro* regarding minimal doubling time (data not shown and [10]). This suggests that specific tumor cell characteristics and/or interactions with the microenvironment are the major determinants causing the significant differences of tumor aggressiveness *in vivo*.

### Genes differentially expressed in fast- versus slow-growing melanoma models are non-randomly distributed along the chromosomes

**Figure 2:**
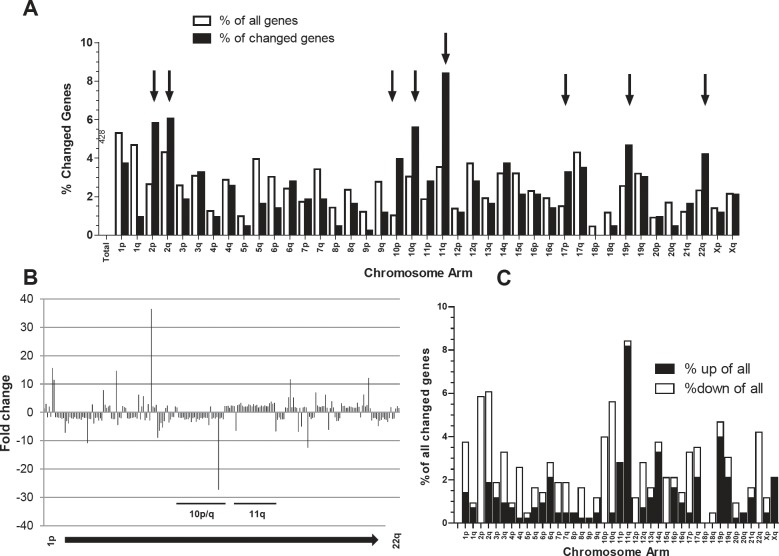
Differentially expressed genes (Student`s t-test, p<0.01; N=428 probes) in the “fast” versus the “slow” melanoma subgroups are not randomly distributed across the chromosomes (A) Percentage of all genes located at the indicated chromosome arms as compared to the percentage of changed genes are indicated by open and black bars, respectively. Chromosome arms with highly increased aberration ratios are indicated (black arrows). (B) Fold changes in the expression levels for the 428 probes in the fast- versus slow-growing subgroup are mapped to the respective chromosomal regions. (C) The percentage of genes significantly up- or downregulated in the fast- versus slow-growing melanoma subgroup at the indicated chromosomal arms were calculated.

In order to determine cellular factors driving *in vivo* tumor aggressiveness, whole-genome gene expression arrays were performed. The 11 melanomas were subgrouped into “fast-growing” and “slow-growing” models according to xenotransplant growth dynamics (compare Figure 1A) in order to extract differentially expressed genes (Student´s t-test p<0.01, 428 oligonucleotide probes representing 323 genes). When allocating this set of probes to the chromosomal arms, a strikingly non-random distribution was detected (Figure 2A,B). First, when comparing the proportion of significantly changed probes per arm with that of all oligonucleotides represented on the microarray, chromosome arms with distinct enrichment of altered gene expression in fast- versus slow-growing melanomas became obvious (Figure 2A). Hotspots were chromosomes 2, 10, 11 and 22 as well as 17p and 19p arms. Also the direction (up- or down-regulation) of the significant gene expression changes was non-randomly distributed along the chromosomes (Figure 2B,C). Thus, for example altered genes on chromosomes 10, 2p, and 22 were almost generally expressed at lower levels in the fast-growing subgroup (39/41; 25/25; 18/18, respectively). In contrast, on chromosome 11 all but one concerned oligonucleotides (47/48) indicated a significantly higher expression in the aggressive melanoma subgroup (Figure 2C). Taken together these data suggest that genomic/chromosomal alterations might have a direct impact on the gene expression pattern associated with in *vivo* aggressiveness of human melanoma models.

### Genomic gains and losses but not mRNA expression patterns cluster with aggressiveness in xenotransplantation models

**Figure 3:**
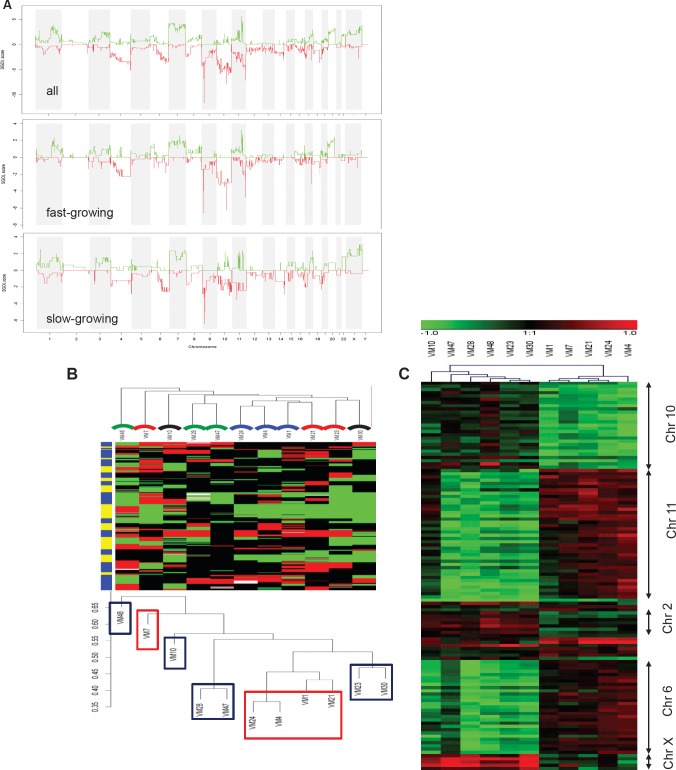
DNA gains and losses analysed by array CGH in melanoma models and association with aggressiveness (A) GISTIC analyses are shown for the 11 melanoma models (upper panel), and for the 5 fast- and the 6 slow-growing models (middle and lower panel, respectively). SGOL scores were calculated using a modified version of the GISTIC algorithm using the “SGOLscore” function (see Material and Methods). (B) Unsupervised clustering (WECCA, see Materials and Methods) of the melanoma models with respect to the array CGH data are shown. “Fast” melanoma models are indicated by red and “slow” ones by blue boxes. Colored arcs indicate the histology/metastatic site of the melanoma models: red, primary nodular melanomas; black, primary superficial spreading melanoma; blue, lymph node metastasis; green, brain metastasis. (C) The heatmap depicts probes (N=120) significantly (p<0.001, Student´s t test) differing in array CGH analysis between the fast- versus slow-growing melanoma subgroups. The predominantly affected chromosomal regions are indicated at the right.

Based on the striking non-random association of genome-wide gene expression with chromosomal regions, we decided to perform array CGH analyses to investigate genome-wide changes in gene copy numbers. All melanoma models exhibited multiple chromosomal changes involving the classical characteristics of human melanoma cells like gains in chromosomes 6p, 7 and 20 as well as losses in 9p, 10 and 14. Array CGH data for the three representative melanoma models VM-4, VM-7 and VM-24 are shown in Supplementary Figure S1. A summary of array CGH data for all 11 melanoma models based on GISTIC analysis is presented in Figure 3A (upper panel). When comparing genomic changes in fast- versus slow-growing melanoma cell models by GISTIC (Figure 3A, middle and lower panel), several differences appeared. While the *p16/ARF* locus at chromosome 9p was equally lost in both subgroups, deletions at the *PTEN* locus at 10q were more pronounced in the aggressive melanomas. Moreover, more extended losses of chromosome 10 and gains of chromosomes 20q and parts of 11q characterised the aggressive melanoma models. In contrast, losses in 6q were more apparent in the less aggressive subgroup.

In order to find out whether genomic changes and/or mRNA expression levels in melanoma models indicate histological origin and/or tumor type, we performed unsupervised cluster analyses on array CGH (Figure 3B) and gene expression data (Supplementary Figure S2). Concerning genomic alterations, clustering of melanoma models reflected neither the histological origin nor the metastatic site, but - with one exception — growth aggressiveness *in vivo*. This was in contrast to the gene expression analyses, where no association with aggressiveness in the mouse models could be found. Upon closer inspection of those genomic regions differing significantly (p<0.001) in gene dose between the two subgroups of melanomas, again a strong prevalence of selected chromosomal arms became visible with a focus on changes in chromosomes 2, 6, 10, 11 and X (Figure 3C).

### Genomic alteration signature predicts aggressiveness of melanoma xenograft models

**Figure 4:**
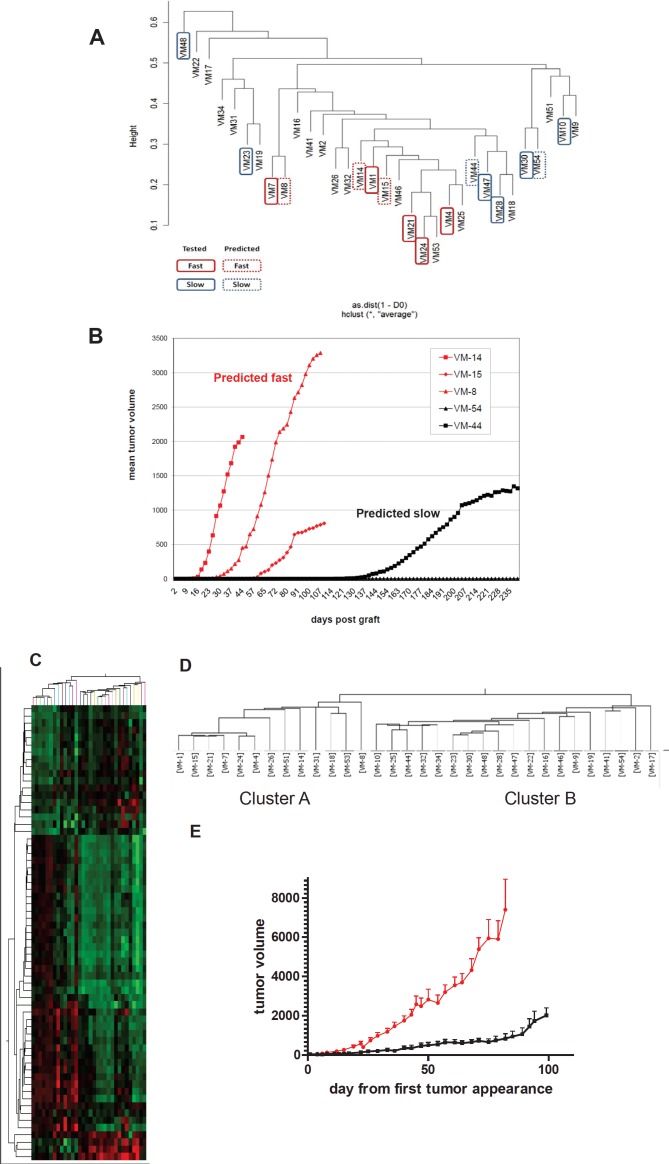
Genomic alterations allow prediction of local growth aggressiveness of human melanoma models in immuno-compromised mice (A) Unsupervised clustering of the original 11 (solid boxes; red and blue indicate fast- versus slow-growing, respectively) and the additional 21 melanoma cell models was performed based on array CGH data. Those melanoma models selected for validation in xenograft growth are indicated by dashed outlines. (B) Xenograft growth characteristics of the melanoma models selected for validation are shown. (C) Clustering of the 32 melanoma models by array CGH data based on probes (N=62; “genomic identifier”) differing at p<0.0005 (Student´s t-test) between the fast- versus the slow-growing subgroup of the original 11 melanoma cell models. The corresponding heatmap and dendrogram are shown. (D) Assignment of the melanoma cell models to the two clusters generated by the genomic identifier probe set. (E) Mean tumor growth from the day of measurable tumor detection of the 9 melanoma xenografts assigned to cluster A (red line) and the 12 xenografts to cluster B (black line) is shown.

To test whether the genomic signature of the original 11 melanoma models allows prediction of aggressiveness in further melanoma models, genomic DNA isolated from 21 additional melanoma primary cell cultures was analysed by array CGH. As a first strategy, unsupervised clustering using the complete array CGH data set was performed in all 32 melanoma models (Figure 4A). Again the previously identified fast-growing cell models tended to cluster together in subgroups (solid-lined boxes) including also novel melanoma models. Consequently, we chose three additional cell models clustering with the fast- and two with the slow-growing subgroup (broken lined boxes in Figure 4A) and tested them for tumor growth in SCID mice. Indeed, a strong difference was found within this “validation set” with VM-8, VM-14 and VM-15 being highly aggressive while VM-44 formed slow-growing tumors and VM-54 was not tumorigenic within the time period of analysis (Figure 4B). In a second approach, we aimed to develop a less complex “genomic signature” from the original 11 melanoma models. Consequently, a highly stringent analysis at the single probe level extracting those probes differing between the two subgroups with a p<0.0005 (Student´s t-test) was performed. This resulted in 62 probes representing 50 annotated gene loci (termed “CGH-identifier”) (Supplementary Table S2). Cluster analyses of all available 32 melanoma models with this CGH-identifier led to formation of two distinct subgroups termed cluster A and B (Figure 4C,D). Consistent with unsupervised clustering results, cluster A (N=13) contained all five fast-growing and cluster B (N=19) all six slow-growing members of the original 11 melanoma models. Of the additional 21 models, 9 grouped in cluster A and 12 in cluster B including correct allocation of the 5 melanoma models used as validation set (compare Figure 4B). The growth dynamics of these melanoma clusters in SCID mice (Fig. 4E) clearly reflected the highly significant difference in tumor growth between the two subgroups. These data indicate that a defined set of chromosomal changes can predict *in vivo* aggressiveness of human melanoma cells.

### Significant gene expression differences between fast- versus slow-growing melanoma models are reflected by gene dose alterations

**Figure 5:**
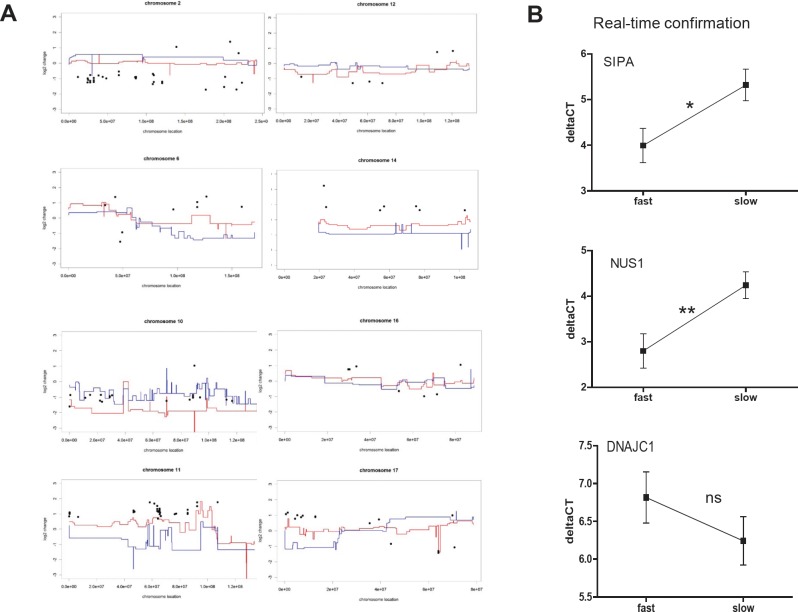
Aneuploidy-driven gene expression deregulation underlies aggressiveness of melanoma xenograft models (A) Genes (191 probes covering 180 genes) differing in mean expression values between the two melanoma subgroups at a significance level of p<0.005 (Student´s t-test, log2 of fold-change, black dots) are blotted for the most informative chromosomes against the mean array CGH aberration scores (log2) for fast- (red lines) and slow-growing (blue lines) melanoma cell models. Accordingly, a value of 1 indicates a two-fold increase and -1 a two-fold decrease in mean expression level in the fast- versus the slow-growing subcluster. In contrast, array CGH data indicate mean (log2) gains/losses in the two melanoma subgroups as compared to normal control DNA pooled from healthy donors. Consequently, a value of 1 indicates a two-fold increased copy number of the respective chromosomal region in the indicated melanoma subcluster. (B) Expression levels of three selected genes from Supplementary Table S2 were evaluated by real-time RT-PCR and mean deltaCT values in the fast- versus the slow-growing subclusters are shown.

Next we re-evaluated gene expression differences between the melanoma subgroups at higher stringency (p<0.005; Student´s t-test). This analysis resulted in 191 probes representing 180 differentially expressed genes. Figure 5 depicts expression changes of these genes (log2 of fold-change, black dots in Figure 5A) plotted for the most informative chromosomes along the mean array CGH aberration scores (log2) for fast- versus slow-growing melanoma subgroups (red and blue lines, respectively, in Figure 5A). A surprisingly good reflection of the expression levels by gene copy numbers was observed for the majority of genes. Only 15.4 % of all 191 significantly changed probes at the mRNA level did not correspond to a respective change at the DNA level. Interestingly, these few genes were mainly localised on three chromosomes/arms, namely 1p, 21q, and X. In contrast, the vast majority or even all significant expression changes at several chromosomal arms were reflected by DNA dose both in terms of gains and losses, like chromosome 2 (29/32 agreeing), chromosome 6 (9/9 agreeing), chromosome 10 (18/19 agreeing), chromosome 11 (28/28 agreeing) and chromosome 17 (14/15 agreeing). This again indicates that large scale gains/losses at several specific chromosomal regions are involved in driving local aggressiveness of melanoma xenograft models.

### Gene expression differences between fast- versus slow-growing melanoma models suggest alterations in cellular growth and differentiation networks

Next we analysed expression array data by in silico pathway prediction approaches for genes expressed differentially between the 8 fast - and 8 slow-growing models (including the original 11 models and the 5 models of the validation set) (Student`s t-test, p<0.05). This resulted in the identification of 2165 altered probes, which were imported into Ingenuity Pathway Analysis software. Regarding biological functions this approach suggested beside “cancer” also both “dermatological” and “neuronal diseases”. Altered networks involved Ras and negative MAPK regulators like Sprouty (Spry) and Spred proteins as well as Ras/Rap/Rab GTPase protein families, suggesting deregulation of these gene products to contribute significantly to melanoma model aggressiveness *in vivo* (Supplementary Figure S3A,B).

### Genes altered at gene dose and expression level between fast- versus slow-growing melanoma models: SIPA1 as example

To identify factors underlying the predictive power of genomic alteration patterns, we aimed to investigate the reflection of gene expression changes in the corresponding gene doses. In order to extract genes or pathways from the genomic signature which determine the observed aggressiveness, again two approaches were followed. First, those genes altered at both the DNA and mRNA level were evaluated with comparably low stringency (genomic level: p<0.01; expression level p<0.05). This analysis resulted in the selection of 116 probes, which were predominantly located on chromosomal regions 11q12-q14, 10p, and 6q. Pathway identification using the Ingenuity Pathway Analysis software revealed several significant pathways involving regulators of GTPases like Rap1 (SIPA1, a RapGAP protein, and RapGEF2) (Supplementary Figure S4), which pointed toward a significant impact of Rap1 deregulation on melanoma model aggressiveness *in vivo*.

In a second approach the evaluation was repeated at higher stringency (genomic level: p<0.005; expression level p<0.005). This approach resulted in only 18 probes/genes that were highly significantly changed both on DNA and mRNA levels (Supplementary Table S3). Within this set of genes, six were expressed at lower levels in the aggressive melanoma models, 5 of which were localized on chromosome 10. Genes expressed at higher levels were predominantly (9 of 12 oligonucleotides) located on chromosome 11q13. In order to validate the genes with the highest significance, real-time PCR experiments for three selected genes were performed. Statistically significantly increased expression of *SIPA1* and *NUS1* in the fast-growing melanoma subcluster was confirmed, while down-regulation of *DNAJC1* was also seen in this analysis but did not reach significance (Figure 5B).

**Figure 6:**
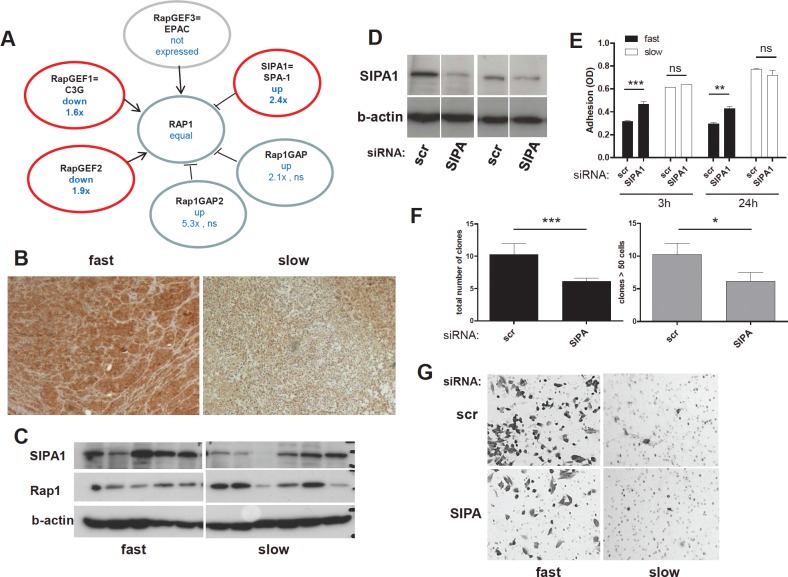
The Rap1 deactivator SIPA1 is gained at the DNA level as well as overexpressed in aggressive melanoma models and impacts on cell behaviour (A) Deregulation of Rap1 activity regulating molecules at the mRNA expression level in the fast- versus the slow-growing melanoma models. Red circles indicate significant changes (p<0.05) and the direction of the changes in fast- versus slow-growing melanoma models is indicated. (B) Immunodetection of SIPA1 in a “fast” (VM-8) and a “slow” (VM-28) melanoma xenograft tumor is shown. (C) SIPA1 and Rap1 expression was detected by Western blot analysis in the original 11 melanoma cell lines grouped with regard to growth aggressiveness. (D) Knock-down of SIPA1 expression was performed using siRNA. Two examples of fast-growing melanoma models are shown. (E) Cell adhesion in a “fast” (VM-1) and a “slow” (VM-28) melanoma cell line transfected with scrambled (scr) and SIPA1 siRNA was determined at 3 h and 24 h after seeding. (F) The impact of SIPA1 knock-down by siRNA on clonogenic potential was measured in the aggressive melanoma cell line VM-1. The total colony numbers (left panel) and the number of large colonies (≥50 cells, right panel) were counted. (G) Impact of SIPA1 siRNA-mediated knockdown on migration potential of “fast” as compared to “slow” melanoma cells was investigated using transwell chamber assays. All experiments were performed three times and means ± SD are shown.

Gene expression analysis did not only indicate significant overexpression of the Rap1-inactivating protein SIPA1 in the aggressive subgroup, but also down-regulation of two RapGEFs and trends towards increased levels of further RapGAP molecules (Figure 6A). Collectively, these data suggest that Rap1 deactivation might represent a key characteristic of locally aggressive melanoma models. To further test the validity of our integrative approach, we chose *SIPA1* which exhibited the strongest alteration among the Rap1-regulatory genes. SIPA1 protein overexpression in fast-growing melanoma cell models was confirmed *in vitro* and *in vivo* (Figure 6B,C). siRNA-mediated gene knock-down (Figure 6D) resulted in significantly enhanced cell adhesion capacity in the fast-growing SIPA1-positive melanoma cell model VM-1 (Figure 6E, “fast”). In contrast, no significant impact was detected in the slow-growing, SIPA1-low melanoma model VM-28 (Figure 6E, “slow”). Furthermore, clonogenic potential and cell migration capacity were reduced by SIPA1 blockade in the aggressive melanoma cell line (Figure 6F,G) while VM-28 cells were almost incapable of migrating through the pores of the trans-well chambers within 48 h (Figure 6G).

## DISCUSSION

Melanoma cells constitutively harbor - besides well-defined mutations in certain proto-oncogenes like *BRAF* and *NRAS* - also non-random genomic alterations reflecting chromosomal instability (CIN) [11, 12]. Furthermore this non-random aneuploidy has been suggested as a major driving force for melanoma development and progression thus representing an attractive entry point for cancer gene discovery [7, 9]. Using *in vivo* growth aggressiveness as a grouping criterion, we provide strong evidence that aneuploidy-mediated gene expression alterations are key drivers of aggressive melanoma growth. Our observations show that 1) genes exhibiting significantly altered expression levels in fast- versus slow-growing tumors are non-randomly distributed along the chromosomes; 2) aggressiveness-associated expression differences reflect copy number alterations at the corresponding DNA loci in the vast majority of affected genes; 3) unsupervised approaches based on array CGH data indeed cluster melanoma models according to aggressiveness; 4) the pattern of DNA alterations consequently allows prediction of *in vivo* growth behavior; 5) genes with different DNA dose and mRNA expression levels include several well-known cancer or even melanoma genes (*PTEN, NRAS, AURKA, ING3*) [5, 11, 13, 14]. Altered gene expression patterns support activation of several oncogenic signaling pathways in the aggressive subgroup including fibroblast growth factor receptor (FGFR) signaling (upregulation of *FGF1* and *FGFR1*, downregulation of *SPRY4* and *SPRED2*), MAPK and PI3K signaling (upregulation of *HRAS* and *NRAS*, downregulation of *PTEN*), and deregulation of small G proteins with a focus on Rap1 family members (upregulation of *SIPA1*, downregulation of *RapGEF1* and *RapGEF2*), mitosis effectors (*AURKA, INCENP*) and invasion/adhesion regulation (*ITGAV, MMP9, MMP19*).

To the best of our knowledge, this is the first study combining integrative genomics with *in vivo* growth aggressiveness as differentiation parameter to identify mechanisms and genes driving local melanoma growth aggressiveness. However, several other studies have used integrative genomic approaches to identify key factors driving melanoma development and/or progression. In agreement with our findings, these reports support the importance of genomic gains/losses and aneuploidy as driving forces in malignant transformation and progression [7]. Already early studies have demonstrated that the degree of aneuploidy and allelic loss might predict unfavorable prognosis [15]. Several specific chromosomal alterations (e.g. loss of chromosomes 6q and 10q, gains in chromosomes 7, 11q and 20q) were demonstrated to be associated with a more malignant phenotype and shorter patient survival [16-18]. Genome-wide array CGH approaches have been used to discriminate between nevi and melanoma [19], and specific genomic alterations were found to be associated with histological subtypes of melanoma [6], *BRAF* mutations [20], anatomical site as well as pattern of UV radiation exposure [19].

The melanoma models used in our study were derived from both primary and metastatic sites (including skin, lymph node, and brain) of both nodular and superficial spreading melanomas and almost generally harbored mutations in either *BRAF* or *NRAS* genes. Interestingly, unsupervised clustering of array CGH data in our melanoma set did not result in subgrouping according to histological subtype or stage of disease but rather reflected aggressiveness of *in vivo* growth behavior. This implies that gene dose is a major player in the deregulated expression of specific genes involved in melanoma progression. Corroborating observations were published e.g. using melanoma models from different species [21] or by comparing primary and metastatic lesions [22]. In a study by Lin et al., unsupervised clustering of 101 melanoma cell cultures based on genomic alterations led to formation of subgroups according to e.g. *BRAF* and *NRAS* status as well as losses at chromosome 10q [23]. In line with our study, multiple genes encoded in the GISTIC-positive regions were demonstrated to be deregulated at the expression level by SAM analysis. Besides the known *BRAF* mutations and *PTEN* loss also an important impact of mutations in *FGFR1* and deregulation of negative MAPK feedback molecules like Spry proteins were detected [23]. Using a Bayesian network-based computational framework on the identical data set, Akavia et al. have recently identified two malignancy driver genes including one Rab GTPase protein [24]. Of note, also in our study upregulation/gain of *FGFR1* (together with *FGF1*), loss of negative MAPK regulators including *SPRY1, SPRY2* and *SPRED2*, and deregulation of multiple small GTPases including Rab- and Rap-regulators were associated, in addition to *PTEN* loss, with a more aggressive melanoma phenotype.

With regard to the intracellular signaling modules indicated to be deregulated in the aggressive phenotype, several alterations like upregulated HRAS and NRAS levels suggest activation of downstream signal modules like the PI3K and the MAPK pathways. Significantly decreased PTEN expression and gene dose in our fast-growing melanomas indicate the necessity for a robust up-regulation of AKT downstream signals as driver for melanoma aggressiveness. Indeed, the PI3K/AKT/mTOR pathway is aberrantly activated in up to 70% of melanomas and has been implicated in tumor progression and chemoresistance. Accordingly, PTEN is inactivated in a high proportion of melanomas through diverse mechanisms [25, 26].

Loss of negative MAPK regulators like Spry and Spred proteins suggests concerted hyperactivation of ERK as a driver of melanoma aggressiveness. Considering the wide-spread activating mutations in *BRAF* this observation was somewhat surprising especially as MAPK signal attenuation by Spry2 was suggested to be lost in melanoma cells harboring *BRAF^V600E^* [27]. Additionally, we found no convincingly enhanced levels of ERK phosphorylation of the fast- as compared to the slow-growing subgroup (data not shown). This argues for a regulatory role of Spry and Spred proteins in melanoma aggressiveness independent of oncogenic BRAF-driven ERK hyperactivation. Down-regulation of Spry proteins might also enhance melanoma aggressiveness by supporting PI3K pathway activation. Thus, Edwin et al. showed that Spry2 upregulated PTEN expression and blocked EGF-mediated AKT activation and cell cycle progression [28]. Accordingly, Spry2 expression was decreased with colon cancer disease progression, and re-expression increased PTEN levels and suppressed growth and migration [29]. Similar to Spry, also Spred proteins act as inhibitors of the MAPK pathway. Spred overexpression has been shown to inhibit cancer motility, metastasis and Rho-mediated actin reorganization [30].

Mining of DNA and mRNA array data from our melanoma models suggested that changes leading to Rap1 deactivation might support the locally aggressive phenotype. For instance, expression of two Rap1 activators (*RapGEF1* and *RapGEF2*) was significantly down-regulated in the fast-growing subgroup. Among the deactivators, the RapGAP *SIPA1* was significantly overexpressed and gained at the DNA level while two further RapGAPs (*Rap1GAP1* and *Rap1GAP2*) were upregulated up to >5-fold without reaching statistical significance. *RAP1* was originally identified as a gene able to reverse the malignant features of KRAS transformed fibroblasts [31]. However, deregulation of Rap1 via Rap1GEFs and Rap1GAPs might have more complex and even opposite impacts on tumor aggressiveness (reviewed in [32]). In melanoma, previously published data implicate both oncogenic and tumor-suppressive roles of Rap1 and its regulators [33-38]. Rap1 activation via downregulation of RapGAP1 was suggested to support ERK activation (even in *BRAF* mutant melanomas) and migration of melanoma cells *in vitro*. [35, 37]. In contrast, Kobayashi et al. demonstrated that Rap1 upregulation might induce melanoma cell death [34]. Accordingly, a clinicopathological study reported that high Rap1GAP expression might be a useful marker to identify high-risk melanoma [33]. Additionally, ERK activation was demonstrated to be mediated by RAS rather than Rap1 in melanocytes [39]. The Rap1 regulatory gene *SIPA1 (SPA-1)* was most distinctly altered at the DNA and mRNA level in our melanoma subgroups but has not been connected to melanoma before. In human solid tumors, a polymorphism in the *SIPA1* gene causing higher RapGAP activity was associated with high metastatic potential of breast cancer [40], and SIPA1 expression was found to positively correlate with disease progression and metastasis in human prostate cancer [41]. Therefore, we decided to knock-down this protein in melanoma models with fast- and slow-growing signature. Interestingly, this led to enhanced cell adhesion but reduced clonogenic potential and migration exclusively in the fast-growing melanoma model, suggesting a complex role of the SIPA1/Rap1 axis in regulating melanoma growth and invasion.

Taken together our study demonstrates that aneuploidy-driven deregulation of gene expression is one major driver defining the degree of local aggressiveness in human melanoma xenograft models. Thus non-random genomic alterations represent - besides activating gene mutations - an additional mechanism promoting concerted hyperactivation of major growth and survival pathways essential for human melanoma aggressiveness *in vivo*.

## MATERIALS AND METHODS

### Primary cell cultures

Primary melanoma cell cultures were established at the Institute of Cancer Research, Medical University Vienna and the Wagner Jauregg Hospital, Linz, and authenticated as previously described [10, 13, 42] and cultured in growth medium containing 10% FCS and 1% glutamine without antibiotics. Histological classification, origin, *BRAF^V600E^* and *NRAS^Q61^* mutation status of 32 melanoma cell cultures are given in Supplementary Table S1.

### Melanoma xenograft models

Subcutaneous tumor growth was initiated by injection of primary melanoma cells into 6-8 weeks old immunocompromised female mice (2.5 × 10^6^ cells into nu/nu mice; Iffa Credo, Charles Rivers, Arbresle, France for the initial 11 melanoma models; or 1 ' 10^6^ cells for all melanoma models into SCID/BALBc; Harlan Winkelman, Borchen, Germany). Each experimental group contained 5 mice. Body weight and tumor size using a vernier caliper [43] were determined three times per week. All *in vivo* experiments described in the present study were performed on the basis of Authorization (LA1230509) of the Animal Ethics Committee of the Federal Department of Health, Nutritional Safety and Environment (Belgium) or according to the Austrian and FELASA guidelines for animal care and protection.

### Histology and immunohistochemistry

Tumor, lung, liver, kidney, and brain were removed, fixed in buffered formalin and embedded in paraffin for conventional histopathological HE staining. Three H&E-stained slides per organ were analyzed to look for metastases. Additional 5 slides through the whole piece were analyzed when the first screening was negative. For staining of tumor sections for SIPA1, the SPA-1 antibody (B-7, Santa Cruz) was applied using a 1:150 dilution.

### Array genomic comparative hybridization (array CGH)

Tumor-DNA was isolated using the DNA Blood Mini Kit from Qiagen (Valencia, CA). Normal human reference DNA from multiple anonymous male donors was purchased from Promega (G147A, Madison, WI). Array CGH analyses using 4x44K oligonucleotide-based microarrays (Agilent, Santa Clara, CA) were performed according to the manufacturer`s protocol and as described previously [44]. Scanning was performed on a G2505B Micro Array Scanner (Agilent). Feature extraction and data analysis were carried out using the Feature Extraction (version 10.7.3.1) and DNA Analytics software (version 4.0.81), respectively. Array CGH raw 2-channel (red/green) log2-ratios were calculated and exported to Excel spreadsheets. Log2-ratios were used (i) as starting point for GISTIC analysis, (ii) as input for un-supervised clustering of chromosomal regions (WECCA), and (iii) for the supervised cluster analysis and graphical representation of significant loci based on un-segmented probe-level data. (i) GISTIC analysis [45] was performed using the GenePattern analysis platform at the public server of the Broad Institute (http://www.broadinstitute.org/cancer/software/genepattern). Segmentation was done employing the CBS algorithm (GenePattern). SGOL scores (Segment Gain Or Loss) were calculated using a modified version of the GISTIC algorithm by the “SGOLscore” function in the “cghMCR” package for Bioconductor 2.5 using R version 2.12.0 [46]. (ii) Unsupervised clustering segmentation and copy number aberrations were calculated by the “CGHcall” algorithm employing the “CGHraw” and “CGHcall” packages for Bioconductor [47]. The algorithm implemented in the “CGHregions” package was used for dimensionality reduction of the region data [47] prior to clustering by the WECCA (Weighted Clustering of Called Array CGH data) method [48]. Cluster representations and dendrograms were generated using the authors’ scripts for R (set to average linkage for agreement). (iii) Graphical representations of supervised clustering of un-segmented probe data were done with Genesis or Genespring. Chromosomal aberration scores were calculated using the Agilent software (ADM-2 algorithm) and plotted in R along the chromosomal positions for the comparisons with mean gene expression changes.

### Whole genome gene expression arrays

Total RNA was isolated by Trizol/Chloroform. Quantity and integrity of the RNA samples was checked on an Agilent 2100 Bioanalyzer (RIN values were >9 in all samples). Gene expression arrays were performed using 4x44K whole genome oligonucleotide-based gene expression microarrays (Agilent). Labeling and hybridization procedures were performed according to the instructions provided by Agilent using the Quick Amp Labeling Kit and the One Colour Microarray-Based Gene Expression Analysis Protocol. Shortly, in a first step 500 ng of total RNA were converted into cDNA using a T7 promoter primer. In a second labeling and amplification step, cDNA was converted into cRNA and labeled with Cy3-CTP. After purification of labeled cRNAs with the RNeasy Mini Kit (Qiagen), 1650 ng per sample were heat fragmentated for 30 min at 60°C. Hybridization was carried out for 17 h at 65°C in a hybridization oven. Afterwards, slides were washed and scanned on a G2505B Micro Array Scanner (Agilent). Feature extraction and data analysis were carried out using the Feature Extraction and Gene Spring software, respectively. Array raw data were also exported to Excel spreadsheets, normalized to the 75^th^ percentile, and used for clustering in the Genesis 1.7.5 software [49]. In cases of redundancy, data have been consolidated by using the probe set with the highest hybridization efficiency. Mean gene expression values (log2-ratios between the fast and slow growing groups) were plotted along the chromosomal positions (hg18 coordinates from Agilent) in R.

### Real-time PCR

Real-time PCR was performed as described [43]. Reactions contained 20 ng cDNA and 20 μl SYBR-Green-Power Mastermix (AppliedBiosystems, California, US) and 0.2 μl per primer. Reactions were set-up in triplicates using the following primers (100 nM): SIPA1, fw 5`-AAG GTG GGC ATC CTG TAC TG-3`, rev 5`-TCT CGT GGT CCT GGT ATG TG-3`; NUS1, fw 5`-CCA GTT AGT AGC CCA GAA GC-3`, rev 5`-GAT GTG CCA GGG AAG AAA GC-3`; DNAJC1, fw 5`-CTC AGC CAA CTG ACA AGA AG-3`, rev 5`-TGA GTT CGG AGA GTC TAA CC-3`; β-actin, fw 5`-GGA GGC AGA AGG AGA TCA CTG-3`, rev 5`-CGA TCC ACA CGG AGT ACT TG-3`; and GAPDH, fw 5`-CTG GCG TCT TCA CCA CCA T-3`, rev 5`-GCC TGC TTC ACC ACC TTC T-3`. PCR was performed on a 7500 Fast Real Time PCR System and results analysed in 7500 Fast System Detection Software (SDS) v1.4 (Applied Biosystems). For the thermal profile of the amplification run, following cycling conditions were chosen: 50°C (2 min), followed by 40 cycles with 95°C (15 s), 60°C (1 min).

### Western blot

Western blot was performed as described previously [13]. The following antibodies were used: SPA-1 (B-7) and RAP1 (both Santa Cruz, CA); β-actin (Sigma, St Louis, MO).

### Suppression of SIPA1 expression by siRNA

5×10^5^ cells were seeded into 6-well plates and incubated for 24 h. Cells were treated with 25 nM SIPA1 siRNA and control scrambled siRNA according to the protocol provided by the manufacturer (DharmaFECT; Dharmacon, Lafayette, CO). After 24 h of incubation with siRNA, cells were counted, and the respective number of cells was seeded and incubated in fresh medium according to the respective procedures.

### Cell adhesion, migration and clonogenic potential

Cell migration and clonogenic capacity of melanoma cells were determined as described in [43] and [50], respectively. For assessment of cell adhesion, cells were treated with siRNA as described above, and 4x10^3^ cells were seeded into 96well plates for 3 h and 24 h. After this time, cells were washed and the medium was removed. After incubation of the remaining adhered cells for 24 h, cell viability was assessed by MTT assay as published [13].

## Supplementary Figures and Tables




