# Supplementary material for: Aggressiveness of human melanoma xenograft models is promoted by aneuploidy-driven gene expression deregulation
Source: Oncotarget. 2012 Apr 24;3(4):399–413. doi: 10.18632/oncotarget.473 (PMC3380575; doi:10.18632/oncotarget.473)
Supplement: Supplementary file 1 [file oncotarget-03-399-s001.pdf]

Aggressiveness of human melanoma xenograft models is promoted by aneuploidy-driven gene expression deregulation – Mathieu et al

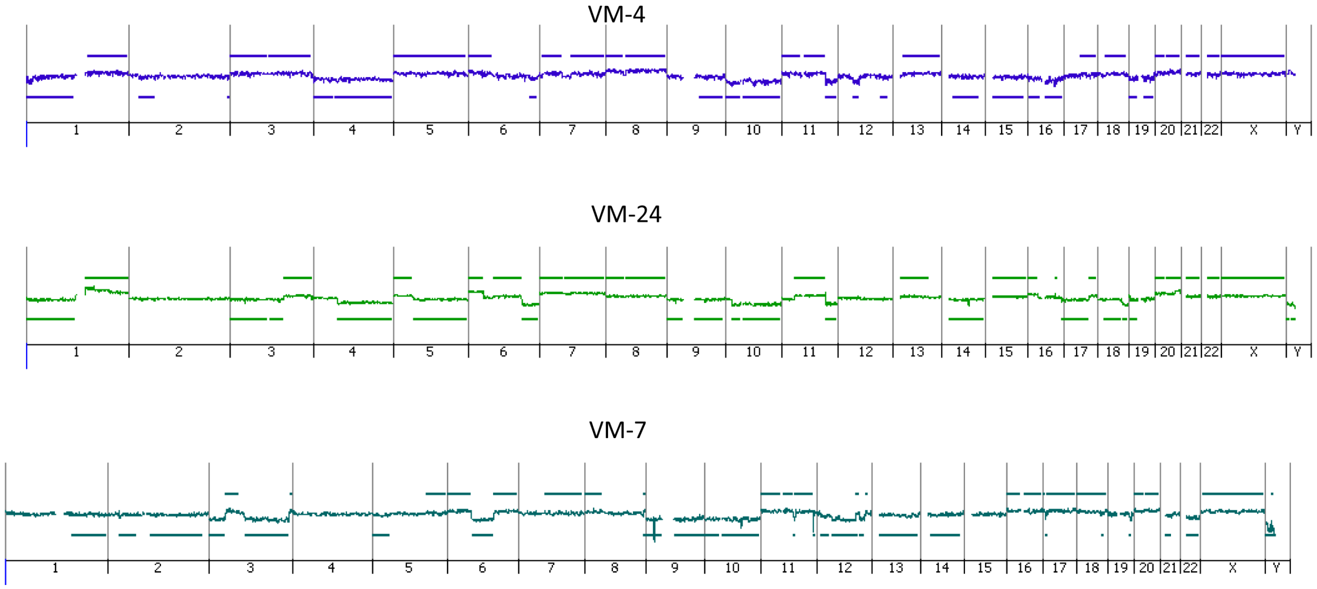

Supplementary Figure S1: Array CGH profiles of three fast-growing melanoma models (algorithm ADM-2, threshold 8, filter default) generated using DNA Analytics software (Agilent).

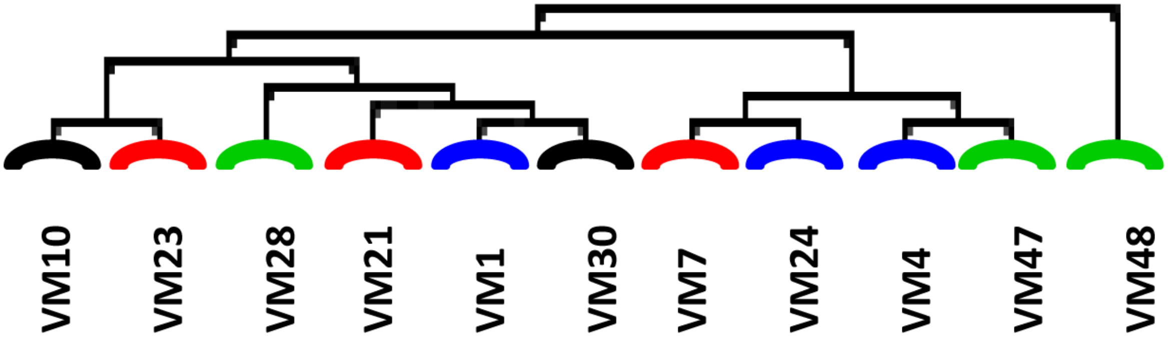

Supplementary Figure S2: Unsupervised cluster analysis of gene expression array data from 11 melanoma xenotransplant models. Unsupervised clustering (WECCA) of the melanoma models with respect to the expression array data is shown. The dendrogram was generated using the authors' scripts for R (set to average linkage for agreement). Colored arcs indicate the histological/organ origin of the melanoma models: red, primary nodular melanomas; black, primary superficial spreading melanoma; blue, lymph node metastasis; green, brain metastasis.

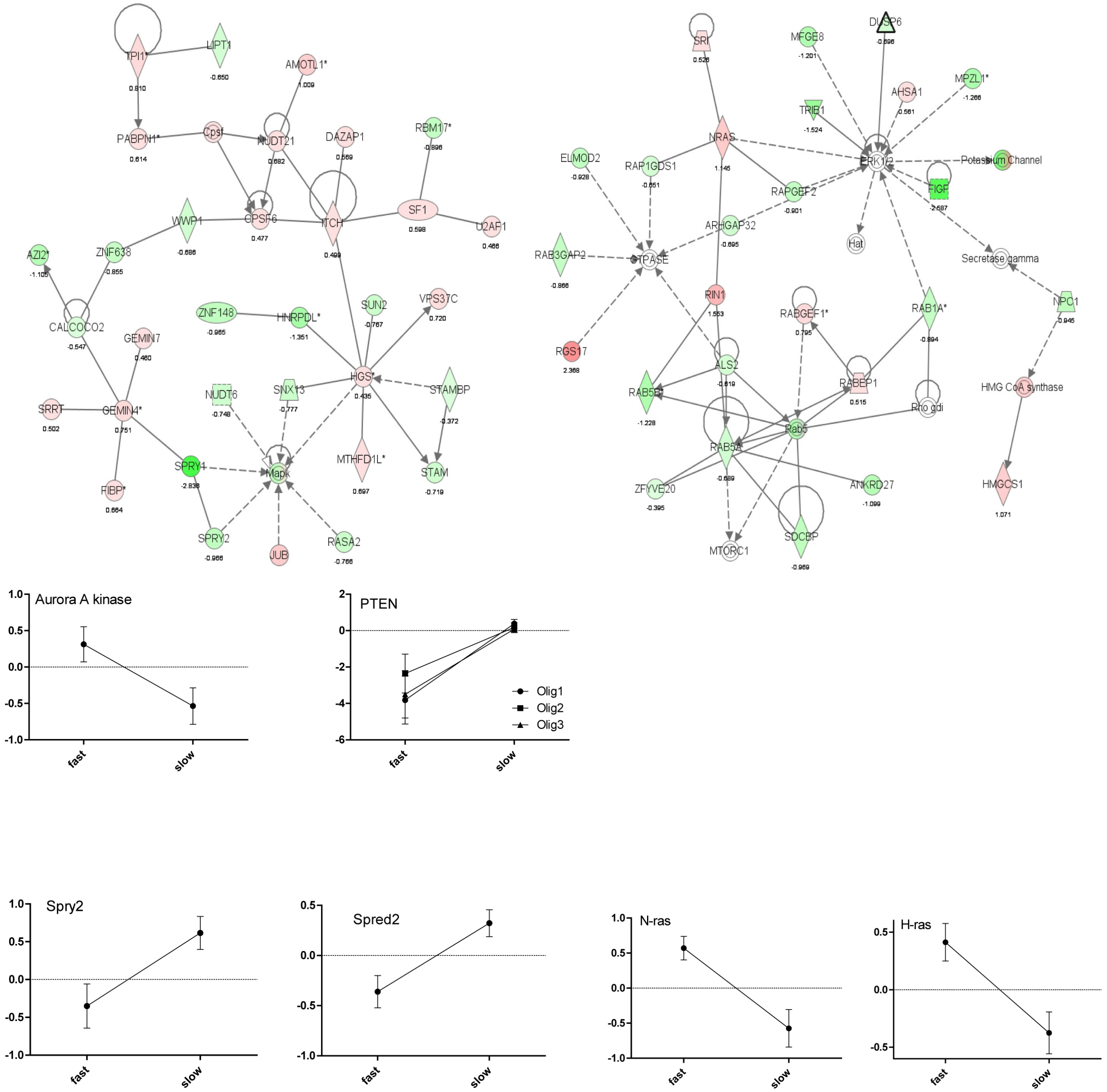

Supplementary Figure S3: (A) Two significantly altered networks between 8 fast- and 8 slow-growing melanoma models as analysed by Ingenuity Pathway Analysis. Red symbols designate upregulated, green symbols downregulated genes. Numbers indicate the fold changes (given as log2) of the respective genes. (B) Expression of the indicated cancer genes analysed by gene expression array are presented in the fast- versus slow-growing melanoma subgroups.

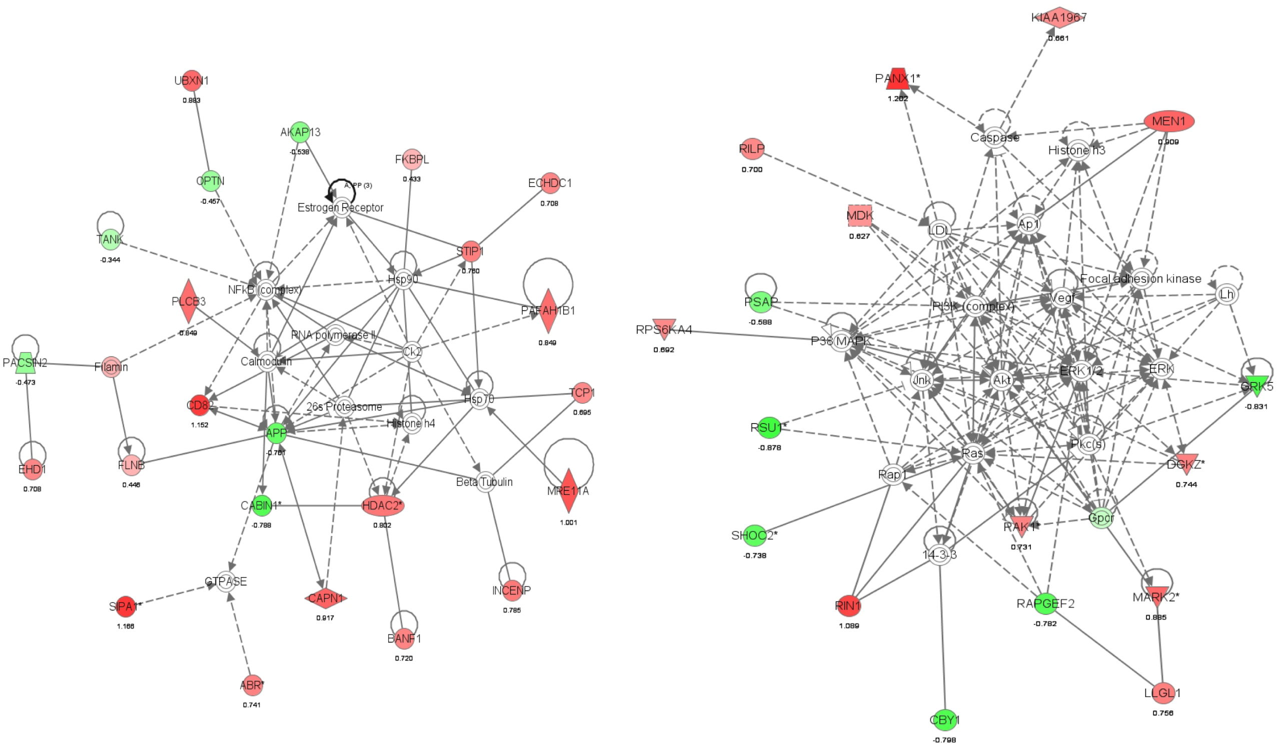

Supplementary Figure S4: Genes (N=116) altered both at the DNA ($p<0.01$) and mRNA level ($p<0.05$) between the fast- versus the slow-growing melanoma subgroups were evaluated by Ingenuity Pathway Analysis. The two pathways altered with highest significance involve the Rap1 regulators SIPA1 and RapGEF2.
